# Comorbidity and cervical cancer survival of Indigenous and non-Indigenous Australian women: A semi-national registry-based cohort study (2003-2012)

**DOI:** 10.1371/journal.pone.0196764

**Published:** 2018-05-08

**Authors:** Abbey Diaz, Peter D. Baade, Patricia C. Valery, Lisa J. Whop, Suzanne P. Moore, Joan Cunningham, Gail Garvey, Julia M. L. Brotherton, Dianne L. O’Connell, Karen Canfell, Diana Sarfati, David Roder, Elizabeth Buckley, John R. Condon

**Affiliations:** 1 Menzies School of Health Research, Charles Darwin University, Casuarina, Northern Territory, Australia; 2 Cancer Council Queensland, Spring Hill, Queensland, Australia; 3 QIMR Berghofer Medical Research Institute, Queensland, Australia; 4 Victorian Cytology Service, Carlton, Victoria, Australia; 5 School of Population and Global Health, University of Melbourne, Melbourne, Victoria, Australia; 6 Cancer Council NSW, Cancer Research Division, Kings Cross, New South Wales, Australia; 7 School of Public Health, University of Sydney, Sydney, New South Wales, Australia; 8 Prince of Wales Clinical School, University of NSW, Sydney, New South Wales, Australia; 9 University of Otago, Wellington, New Zealand; 10 Cancer Epidemiology & Population Health, University of South Australia, Adelaide, South Australia, Australia; Texas Technical University Health Sciences Center, UNITED STATES

## Abstract

**Background:**

Little is known about the impact of comorbidity on cervical cancer survival in Australian women, including whether Indigenous women’s higher prevalence of comorbidity contributes to their lower survival compared to non-Indigenous women.

**Methods:**

Data for cervical cancers diagnosed in 2003–2012 were extracted from six Australian state-based cancer registries and linked to hospital inpatient records to identify comorbidity diagnoses. Five-year cause-specific and all-cause survival probabilities were estimated using the Kaplan-Meier method. Flexible parametric models were used to estimate excess cause-specific mortality by Charlson comorbidity index score (0,1,2+), for Indigenous women compared to non-Indigenous women.

**Results:**

Of 4,467 women, Indigenous women (4.4%) compared to non-Indigenous women had more comorbidity at diagnosis (score ≥1: 24.2% vs. 10.0%) and lower five-year cause-specific survival (60.2% vs. 76.6%). Comorbidity was associated with increased cervical cancer mortality for non-Indigenous women, but there was no evidence of such a relationship for Indigenous women. There was an 18% reduction in the Indigenous: non-Indigenous hazard ratio (excess mortality) when comorbidity was included in the model, yet this reduction was not statistically significant. The excess mortality for Indigenous women was only evident among those without comorbidity (Indigenous: non-Indigenous HR 2.5, 95%CI 1.9–3.4), indicating that factors other than those measured in this study are contributing to the differential. In a subgroup of New South Wales women, comorbidity was associated with advanced-stage cancer, which in turn was associated with elevated cervical cancer mortality.

**Conclusions:**

Survival was lowest for women with comorbidity. However, there wasn’t a clear comorbidity-survival gradient for Indigenous women. Further investigation of potential drivers of the cervical cancer survival differentials is warranted.

**Impact:**

The results highlight the need for cancer care guidelines and multidisciplinary care that can meet the needs of complex patients. Also, primary and acute care services may need to pay more attention to Indigenous Australian women who may not obviously need it (i.e. those without comorbidity).

## Introduction

Cancer patients commonly have comorbidities.[1, 2] There is evidence that higher comorbidity burden is associated with worse survival for cancer patients.[3–5] The prevalence of comorbidity varies between population groups and may contribute to cancer survival disparities, particularly for different ethnic groups.[6–11]

Evidence regarding the effect of comorbidity on survival for cervical cancer is limited and inconsistent: comorbidity was not associated with higher mortality for Italian[12] or Mexican[13] women with cervical cancer; it was associated with higher all-cause mortality but not cancer-specific mortality for women in the United States;[14, 15] and it was associated with both all-cause and cancer-specific mortality for New Zealand (NZ) women.[11]

In Australia, cervical cancer incidence and mortality have fallen by approximately 50% since the introduction of the National Cervical Screening Program in 1991.[16, 17] Five-year survival also improved during the 1990s and has remained steady at about 72% since then.[18] However, cervical cancer has a much greater impact on Aboriginal and Torres Strait Islander women (respectfully referred to hereafter as Indigenous) than other Australian women; semi-national and national analyses suggest Indigenous women are diagnosed younger (median age 46 vs. 52 years), incidence is 2.2 times higher, mortality 3.8 times higher and five-year survival is 20 percentage points lower for Indigenous women (58% vs 78%).[18–20] In Queensland, Indigenous women have lower cervical screening participation than non-Indigenous women (two-year participation 34% vs 56% in 2010–2011)[21] and those who develop cervical cancer are less likely to be diagnosed with localised disease (46% vs. 69%)[22] and receive cancer treatment concordant with clinical guidelines (77% vs 96%).[23]

The prevalence of many chronic diseases including coronary heart disease, diabetes, kidney disease and hypertension is higher for Indigenous Australians compared with other Australians[24] including for women with cervical cancer,[22] but the impact of comorbidity on cervical cancer survival in Australia has not been examined for Australian women generally or Indigenous women specifically. A NZ study found Māori and Pacific women diagnosed with cervical cancer had more chronic disease and excess cervical cancer mortality (i.e. lower survival) compared with other NZ women.[11] While adjusting for comorbidity as a summary measure did not reduce the excess mortality, adjusting for 12 individual chronic conditions appeared to reduce the excess by 21% for Māori and 35% for Pacific women.[11]

Our aims were to investigate whether: (1) comorbidity is associated with lower cervical cancer survival for Australian women generally; (2) higher comorbidity burden contributes to lower survival for Indigenous women; (3) the effect of comorbidity on survival varies by type of comorbid condition; and (4) comorbidity is associated with more advanced stage of disease at diagnosis.

## Methods

We linked cancer registry data for cases of cervical cancer (ICD-10 C53) with hospital inpatient data to obtain comorbidity information. Data were available from six Australian states/territories: New South Wales (NSW); Victoria (Vic); Queensland (Qld); South Australia (SA); Western Australia (WA); and the Northern Territory (NT); together covering 96% of the total Australian female population and 95% of the Indigenous female population.[25] Cases from all jurisdictions entered the study in January or July 2003, except for those in Victoria who entered the study in January 2007 because the Victorian Cancer Registry does not have adequate cause of death data before 2007. To maximise the data available, cases were included if they were diagnosed from study entry up until December 2007 (NSW), 2009 (Qld), 2010 (NT), 2011 (WA) and 2012 (Vic and SA). Cases were followed-up until December in the following year (e.g. for NSW this was December 2008).

Cancer registry data included: Indigenous status; date of birth; place of residence (postcode for Vic and WA, statistical local area for other jurisdictions); date of diagnosis; histological type of cancer; date of death; cause of death; and, for NSW only, summary stage at diagnosis, classified as localised, regional, distant metastases, or unknown/missing stage. Indigenous status is not included in most pathology reports, which are the primary source of notifications to cancer registries. Registries obtain Indigenous status from other notification sources including hospital inpatient episodes, radiotherapy clinics, death notifications, active follow-up with treating doctors, and other secondary sources. The national standard Indigenous identification question asks whether the person identifies as: Aboriginal; Torres Strait Islander; both; or neither. For most Australian states, Indigenous identification in hospital data is considered to be of high-quality. Nationally, 88% of Indigenous patients were estimated to be correctly identified in Australian public hospitals in 2011–12. For states included in this study, accuracy ranged from 78% in Victoria to 98% in the Northern Territory during this time[26]

Death data were not available for cases who had died outside of Australia and it is unknown how many cases are missed due to this. Ascertainment of deaths by the cancer registries was complete for deaths occurring up to the end of the follow-up period in each jurisdiction. Most registries provided only month and year for date of birth, diagnosis and death, so the 15^th^ of the month was used to calculate survival time and age at diagnosis for all cases. Survival time was calculated as the number of months from diagnosis to death or the end of follow-up. Cause of death was classified as cervical cancer or other. Histological type of cancer was grouped as: squamous cell carcinoma; adenocarcinoma; adenosquamous carcinoma; and other carcinoma or sarcoma.[16] Mapping to place of residence was done using the Statistical Local Area (SLA) definitions of the 2006 Australian Geographical Classifications (ASGC)[27] or using postcode information (for Vic and WA). SLA or postcode of residence was mapped to the Socio-economic Index for Areas (SEIFA) 2006 Index of Relative Socioeconomic Advantage and Disadvantage (IRSAD) and divided into five quintiles (‘most advantaged’ to ‘most disadvantaged’),[28] and to the Accessibility/Remoteness Index for Australia (ARIA) 2006 and classified into five remoteness categories (‘major city’ to ‘very remote’).[29]

### Record linkage

In each jurisdiction, the data custodians of the cancer registry and hospital inpatient dataset provided identifying information (name, date of birth, place of residence) to their local data linkage unit (DLU) or Health Department who performed the matching and returned project-specific linkage keys to the custodians. The custodians then provided de-identified clinical datasets to the research team who merged the two datasets using the linkage keys. Each jurisdiction’s linked data was then combined into the semi-national dataset for analysis. The linkage process for Queensland has been described in detail previously.[30] Three states (NSW, Vic, WA) provided data for both private hospital and public hospital admissions and three jurisdictions (Qld, SA, NT) provided data for public hospital admissions only.

### Eligibility criteria

For each woman, the first eligible cervical cancer diagnosis within the study period was included. Women were excluded if they were diagnosed: with a micro-invasive cervical cancer; on the basis of death certificate only, autopsy only, or basis unknown; younger than age 22 years (to allow for sufficient measurement of baseline comorbidity); or older than 89 years (because assignation of cause of death is less reliable for this age-group[31, 32]). In Queensland, cervical cancer diagnoses were provided for women aged up to 69 years only.

### Indigenous status

Women were classified as Indigenous if they were recorded as Indigenous (Aboriginal and/or Torres Strait Islander) in the cancer registry or in at least half of their linked inpatient admissions during the entire study period (as per an algorithm we have used previously[30]).

### Comorbidity

Comorbidity at the time of cancer diagnosis was measured using both the Charlson Comorbidity Index (CCI)[33] and the Elixhauser index.[34] Inpatient episodes with an admission date up to 730 days (two years) prior to, and including, the date of cancer diagnosis were searched for primary and secondary diagnosis (ICD) codes for the conditions included in each of the indices (excluding gynaecological cancers), using validated coding algorithms.[35] For each woman, binary indicators were created for each condition (present/absent), which were used to calculate the Charlson score (a weighted sum of the number of conditions present[33]) and the Elixhauser score (an unweighted count of the number of conditions present[11]). The measures were then categorised as score ‘0’ (no known comorbidity), ‘1’ and ‘2+’, with higher scores indicating higher comorbidity burden.

### Analysis

The difference in median age at diagnosis (which was positively skewed) for Indigenous and non-Indigenous women was tested using the Mann-Whitney U-test. Other clinical and demographic characteristics of Indigenous and non-Indigenous women were described using frequency distributions and compared using the Pearson’s chi-squared test. Cause-specific and all-cause survival were estimated by unadjusted Kaplan-Meier survival probabilities, stratified by comorbidity score, Indigenous status, and age group.

Multivariable regression analysis of five-year cause-specific and all-cause mortality was performed using flexible parametric models. Using the Bayesian Information Criterion, four knots (three degrees of freedom), placed at default locations of survival time, were determined to provide the best fit to model the baseline hazard function. The effect of comorbidity on survival varied by month after diagnosis (p<0.01). This meant that the conventional Cox proportional hazards regression could not be used as the assumption of proportionality was violated. A term was added to the flexible parametric model to account for the time-dependent effect of comorbidity. The main effects model included variables: Charlson score (0,1,2+); Indigenous status; age at diagnosis (in years); quintiles of area-level socioeconomic advantage and disadvantage; and cervical cancer histology type. The associations with diagnosis period, state/territory and place of residence were found to be small and not statistically significant (likelihood-ratio chi-squared test p>0.1), so these variables were not included in the model. The association between comorbidity and the survival differential between Indigenous and non-Indigenous women was initially assessed by comparing the hazard ratio from models with and without comorbidity and examining whether the corresponding confidence intervals overlapped. Likelihood ratio chi-squared tests were used to assess if one model was a better fit than another. Interactions between comorbidity and all confounders and Indigenous status and all confounders were examined during model development and inclusion was based on effect size and statistical significance (Wald test p<0.01). Interaction terms for comorbidity by Indigenous status and comorbidity by age at diagnosis were subsequently included in the final model, along with the variables included in the main effects model.

The main effects model and the final model with interactions were fitted using different measures of comorbidity: (1) the Elixhauser score; (2) indicator variables for each comorbid condition, one at a time; (3) all Charlson indicator variables found to be associated with mortality in the previous models (HR>1.5; a previously used cut-point[11]); and (4) all Elixhauser indicator variables found to be associated with mortality (HR>1.5).

### Relative survival

Relative survival requires population denominator data (life tables). Life tables stratified by comorbidity level are not available and life tables stratified by Indigenous status are only available for limited years. We used cause-specific survival, but this method relies on accurate cause of death data to distinguish deaths caused by cervical cancer from deaths due to other causes. To support the use of this method, we compared cause-specific with relative survival for Indigenous and non-Indigenous women diagnosed during 2006–2010, without adjusting for comorbidity level.

### Sensitivity analyses

This study assumed women with cervical cancer who did not link to a hospital record had no comorbidity. Sensitivity analyses were performed to compare the general patterns of survival and mortality when women who did not link to a hospital record (over the entire period for which data was provided) were included and excluded from the analysis. This was done using Kaplan-Meier survival estimates and flexible parametric survival models, as described above.

### Sub-group analysis for stage of cancer

For NSW cases (24% of total cases), logistic regression was used to assess associations between stage at diagnosis and Charlson score and Indigenous status, separately. Multivariable flexible parametric survival models were used to assess the association between Charlson score and cause-specific mortality, comparing models excluding and including stage at diagnosis, and adjusted for age at diagnosis and Indigenous status. Finally, an interaction term for comorbidity and cancer stage was included in this model to predict and graph five-year survival for women with and without comorbidity, stratified by stage at diagnosis.

All analyses were conducted in Stata 14 (StataCorp, College Station, Texas, USA). For flexible parametric survival models, the *stpm2* package of commands was used.

### Ethics and approvals

The study was approved by 13 Human Research Ethics Committees (HRECs), including specialist Indigenous HRECs, as well as data custodians and data linkage units in each participating jurisdiction. Anonymised data was provided only, which meant it was not possible to contact women for consent and the requirement of individual consent was waived by the ethics committees.

## Results

Of 4,467 women eligible for inclusion, Indigenous women were: younger at diagnosis; more likely to live in remote areas and areas of socio-economic disadvantage; and less likely to be diagnosed with an adenocarcinoma (Table 1). Prevalence of any comorbidity was higher for Indigenous than non-Indigenous women (Charlson: 24.2% vs. 10.0%; Elixhauser: 45.4% vs 16.6%, respectively) (Table 2). The most common conditions for Indigenous women included: excessive alcohol use (13%); uncomplicated hypertension (11%); fluid and electrolyte disorders (9.1%); complicated (8%) and uncomplicated diabetes (7%); deficiency anaemia (6%); serious kidney disease (6%); and chronic respiratory disease (6%) (Table 2 and S1 Table). While more comorbidities were identified using the Elixhauser score, the association between comorbidity and cause-specificr survival was similar regardless of which comorbidity measure was used (see S1–S5 Tables). Charlson comorbidity score is reported hereafter, unless stated otherwise.

**Table 1:** Demographic and clinical characteristics of 4,467 Australian women diagnosed with cervical cancer 2003–2012, by Indigenous status.

	Indigenous	Non-Indigenous	*P*^a^
TOTAL n(%)	198(4.4)	4,269 (95.6)	
Age in years (median, IQR)	41.6 (35.2, 51.1)	47.0 (36.8, 61.4)	<0.001
Age group	**%**	**%**	
22–39 years	44.4	32.8	<0.001
40–49 years	28.3	23.9	
50–59 years	16.2	17.6	
60–69 years	7.1	12.8	
70–79 years	3.5	6.7	
80–89 years	0.5	6.1	
State/territory^b^			
New South Wales	20.7	24.1	<0.001
Victoria	8.1	24.8	
Queensland	27.8	20.9	
South Australia	2.5	11.2	
Western Australia	26.8	17.9	
Northern Territory	14.1	1.1	
Area-level socioeconomic disadvantage quintile (Q)			
Most advantaged(Q5)	3.5	19.8	<0.001
Q4	19.7	26.0	
Q3	20.7	27.5	
Q2	20.2	14.6	
Most disadvantaged (Q1)	32.3	11.4	
Missing	3.5	0.8	
Place of residence			
Major city	25.3	72.6	<0.001
Inner regional	17.2	16.4	
Outer regional	24.8	8.6	
Remote	14.7	1.4	
Very remote	15.7	0.2	
Missing	2.5	0.8	
Histological type of cervical cancer			
Squamous cell carcinoma	78.3	65.2	<0.001
Adenocarcinoma	10.1	23.9	
Adeno-squamous carcinoma	2.0	3.1	
Other carcinoma or sarcoma	9.6	7.8	
Stage of cancer at diagnosis, *For 1*,*069 NSW women only*	*41 women*	*1*,*028 women*	
Localised	41.5	47.2	0.09
Regional spread	34.2	27.8	
Distant metastases	17.1	8.3	
Unknown	7.3	16.7	

Abbreviations: IQR: Interquartile range; Q: Quintile; NSW: New South Wales; P: p-value

NOTES:

a. Mann-Whitney U-test was used to test differences between medians; Chi-squared test was used to test differences in proportions. b. In all jurisdictions, except Queensland, data were provided for women diagnosed with cervical cancer aged 22–89 years. In Queensland, data were provided for women up to age 69 years only.

**Table 2:** Prevalence of conditions in the Charlson Comorbidity Index (%) for Australian Indigenous and non-Indigenous women aged 22–89 years diagnosed with cervical cancer, 2003–2012.

	Indigenous (n = 198) %	Non-Indigenous (n = 4,269) %	*P*^a^
Charlson Condition ^b^			
Acute myocardial infarction	1.5	0.6	0.14
Congestive heart failure	4.0	0.9	<0.001
Peripheral vascular disease	0.5	0.5	0.94
Cerebrovascular disease	1.5	0.5	0.07
Dementia	0.0	0.5	0.31
Chronic pulmonary disease	5.6	1.8	<0.001
Connective tissue disease	0.5	0.4	0.77
Peptic ulcer disease	0.0	0.1	0.60
Mild liver disease	2.5	0.7	0.003
Diabetes without complications	6.6	1.7	<0.001
Diabetes with complications	7.6	2.3	<0.001
Paraplegia	1.0	0.3	0.12
Moderate-severe kidney disease	5.6	1.7	<0.001
Invasive cancer, excl. gynaecological	3.5	1.6	0.04
Moderate-severe liver disease	0.5	0.2	0.27
Metastatic cancer, excl. gynaecological	3.0	1.5	0.10
AIDS	0.0	0.0	
Charlson score^b^			<0.001
0 (No known comorbidity)^c^	75.8	90.0	
1	7.6	3.3	
2	5.1	3.0	
3	3.0	0.8	
4+	8.6	2.8	

NOTES:

a. Chi-squared test was used to test differences in proportions. b. Comorbidity was measured using diagnoses codes contained in hospital admissions data for the two years prior to and including the woman’s cervical cancer diagnosis date. c. No known comorbidity includes women who linked to hospital records and did not have comorbidity and women who did not link to a hospital record and have unknown comorbidity.

### Cause-specific mortality

1,171 women died within five years of diagnosis; 82.0% died from cervical cancer. For all women combined, five-year cause-specific survival was 75.8% (Table 3). Five-year survival decreased with increasing levels of comorbidity for non-Indigenous women, yet for Indigenous women survival was similar for those with a Charlson score of 0 or 1 but much lower for those with a score of 2+.

**Table 3:** Crude five-year Kaplan-Meier survival estimates for 4,467 Australian women (22–89 years)^a^ diagnosed with cervical cancer, 2003–2012, stratified by Charlson comorbidity score (0,1,2+).

	**Five-year *cause-specific* survival (%, 95%CI)**			
	**All women**	**Charlson comorbidity score** ^**b**^		
		**0**	**1**	**2+**
All women	75.8 (74.4–77.2)	79.0 (77.6–80.3)	67.4 (58.6–74.7)	36.6 (30.2–43.1)
Indigenous status				
non-Indigenous	76.6 (75.1–77.9)	79.6 (78.1–80.9)	66.9 (57.6–74.6)	37.3 (30.4–44.1)
Indigenous	60.2 (52.4–67.0)	64.8 (56.1–72.2)	72.0 (41.2–88.6)	32.2 (14.7–51.1)
Age group				
22–39 years	88.9 (87.0–90.5)	89.6 (87.7–91.1)	82.4 (54.7–93.9)	41.9 (18.3–64.1)
40–49 years	81.3 (78.5–83.7)	83.1 (80.3–85.5)	66.8 (46.1–81.0)	47.8 (30.8–62.9)
50–59 years	70.2 (66.5–73.6)	73.0 (69.2–76.5)	81.3 (63.0–91.1)	32.5 (17.5–48.5)
60–69 years	63.4 (58.8–67.6)	67.3 (62.5–71.8)	59.4 (35.8–76.8)	33.8 (21.0–47.0)
70–79 years	57.9 (51.3–64.0)	61.2 (53.3–68.1)	55.6 (32.2–73.8)	46.9 (31.9–60.5)
80–89 years	38.2 (31.1–45.3)	42.1 (33.6–50.3)	60.1 (35.7–77.8)	14.8 (4.0–32.1)
Diagnosis period				
2003–2007	75.6 (73.8–77.3)	79.1 (77.3–80.8)	64.9 (54.2–73.7)	37.1 (29.3–44.9)
2008–2012	76.3 (73.8–78.6)	78.9 (76.4–81.2)	73.1 (56.7–84.1)	36.9 (26.2–47.7)
	**Five-year *all-cause* survival (%, 95%CI)**			
	**All women**	**Charlson comorbidity score** ^**b**^		
		**0**	**1**	**2+**
All women	70.8 (69.4–72.3)	75.3 (-73.8–76.8)	52.3 (43.6–60.3)	24.3 (19.3–29.7)
Indigenous status				
non-Indigenous	71.7 (70.2–73.1)	75.9 (74.4–77.3)	52.3 (43.0–60.7)	24.9 (19.5–30.6)
Indigenous	53.4 (45.8–60.4)	61.0 (52.2–68.6)	52.5 (25.2–74.0)	20.2 (8.0–36.4)
Age group ^a^				
youngest-39 years	87.6 (85.7–89.3)	88.3 (86.4–90.0)	82.4 (54.7–93.9)	39.5 (17.2–62.2)
40–49 years	78.3 (75.4–80.9)	80.5 (77.6–83.1)	59.9 (39.5–75.4)	42.2 (26.8–56.8)
50–59 years	65.7 (61.9–69.2)	70.2 (66.2–73.8)	66.6 (46.4–80.6)	22.9 (11.9–36.1)
60–69 years	57.6 (53.1–61.9)	62.8 (57.8–67.4)	41.6 (20.7–61.3)	25.4 (14.9–37.2)
70–79 years	44.9 (38.7–50.9)	50.6 (43.0–57.7)	48.2 (26.6–66.8)	24.5 (14.2–36.3)
80–89 years	21.3 (16.1–27.0)	27.1 (20.3–34.3)	19.8 (6.3–38.6)	3.2 (0.3–12.2)
Diagnosis period				
2003–2007	70.3 (68.4–72.1)	75.5 (73.6–77.2)	49.2 (39.0–58.7)	23.3 (17.5–29.6)
2008–2012	72.1 (69.5–74.5)	75.3 (72.7–77.8)	61.6 (45.8–74.1)	28.6 (19.4–38.4)

Abbreviations: CI: Confidence Interval

Notes:

a. All jurisdictions provided data for women aged 22–89 years at diagnosis of cervical cancer, with the exception of Queensland, which only provided data for women aged 22–69 years at diagnosis. b. No known comorbidity (0) includes women who linked to hospital records and did not have comorbidity and women who did not link to a hospital record and have unknown comorbidity.

In multivariable analysis, without interaction terms, the age-adjusted risk of dying was 2.6 times higher for Indigenous than non-Indigenous women (HR 2.6, 95%CI 2.0–3.3; S6 Table). The hazard ratio reduced when adjusted for histology type and socioeconomic status (HR 2.2, 1.7–2.8; LR test p<0.001 compared to the previous model), and reduced further when adjusted for comorbidity score (HR 1.8, 1.4–2.3; LR test p<0.001). Ten of the Charlson conditions had HRs >1.5 in separate models (S7 Table), but adjustment for each of these individual conditions (rather than the summary Charlson score) resulted in a similar adjusted Indigenous: non-Indigenous hazard ratio (HR 2.0, 1.5–2.6, LR test P<0.001).

**Fig 1:**
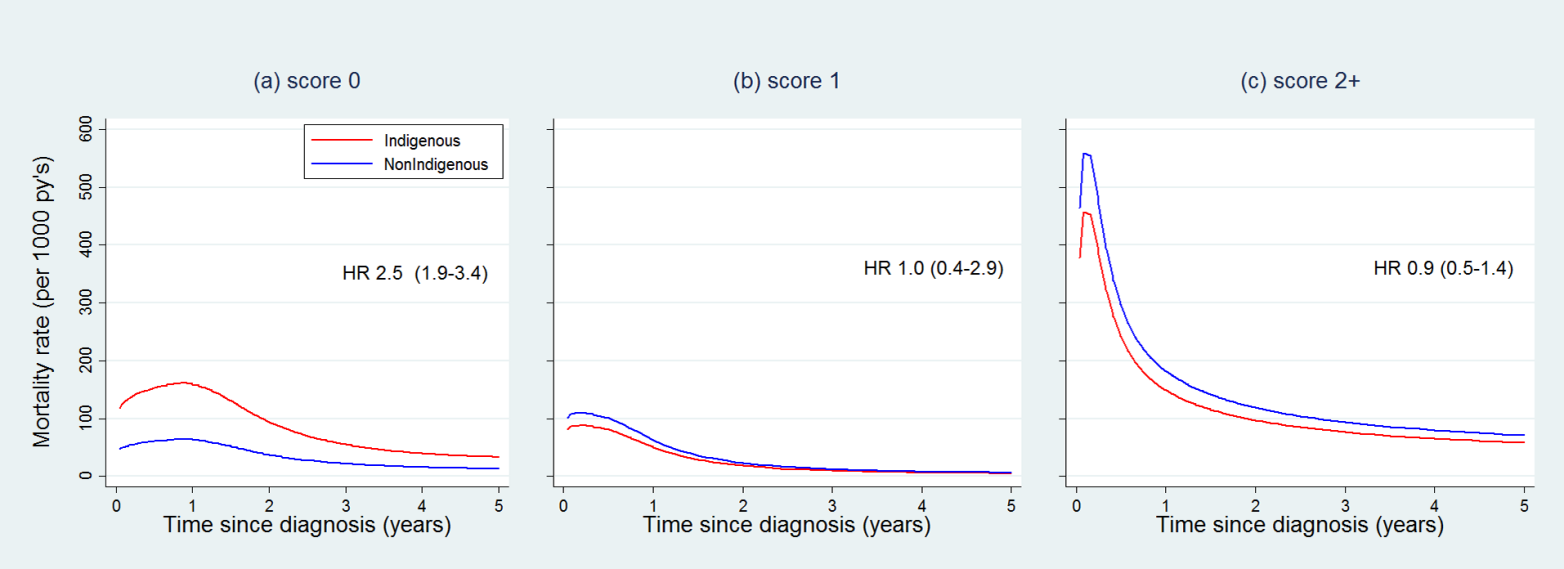
Cause-specific mortality rates for Indigenous and non-Indigenous women stratified by Charlson Comorbidity Index score, adjusted for age at diagnosis, area-level socioeconomic status, and histology type.

However, the relationship between comorbidity and survival differed for Indigenous and non-Indigenous women and by age at diagnosis. When interaction terms were added to the multivariable model, mortality increased significantly with increasing comorbidity only for non-Indigenous women (Table 4). For Indigenous women, these differences were not statistically significant (Table 4). These patterns meant the mortality differential for Indigenous and non-Indigenous women was only evident for those without comorbidity (Fig 1). Increasing age at diagnosis was strongly associated with higher mortality for women without comorbidity (4% increase in mortality per year of age), but the association was smaller for women with comorbidity score of 1 or 2+ (and confidence intervals bordered 1.0) (Table 4).

**Table 4:** Adjusted hazard ratios for five-year cause-specific mortality for Australian women, 22–89 years, diagnosed with cervical cancer, 2003–2012 ^a, b^.

	Adjusted HR ^c^	95%CI
**Charlson comorbidity score**	Chi-sq = 15.8, df = 2, p<0.001^e^	
*Indigenous women*		
0 (No known comorbidity) ^d^	1.00	
1	0.68	(0.24–1.89)
2+	1.57	(0.91–2.70)
*Non-Indigenous women*		
0 (No known comorbidity) ^d^	1.00	
1	1.72	(1.10–2.68)
2+	4.62	(3.54–6.03)
**Age at diagnosis, per year increase**	Chi-sq = 24.46, df = 2, p<0.001^e^	
Charlson score 0 ^d^	1.04	(1.04–1.04)
Charlson score 1	1.02	(1.01–1.04)
Charlson score 2+	1.01	(1.00^f^-1.02)
**Area level socioeconomic disadvantage**	Chi-sq = 24.9, df = 5, p<0.001^g^	
Most advantaged (Q5)	1.00	
Q4	0.96	(0.78–1.18)
Q3	1.15	(0.94–1.40)
Q2	1.33	(1.07–1.65)
Most disadvantaged (Q1)	1.51	(1.20–1.89)
Missing	0.79	(0.40–1.57)
**Histology type**	Chi-sq = 75.4, df = 3, p<0.001^g^	
Squamous cell carcinoma	1.00	
Adenocarcinoma	0.77	(0.65–0.92)
Adenosquamous carcinoma	1.24	(0.87–1.76)
Other carcinoma or sarcoma	2.19	(1.81–2.64)

Abbreviations: HR: hazard ratio; CI: confidence interval; Q: quintile.

Notes:

a. All jurisdictions entered the study in 2003, except for Victoria which entered the study in 2007. Jurisdictions exited the study in 2007 (NSW), 2009 (QLD), 2010 (NT), 2011 (WA), and 2012 (VIC and SA). b. All jurisdictions contributed women aged 22–89 years at diagnoses, except for Queensland which contributed women aged 22–69 years only. c. Hazard ratios were mutually adjusted for all variables and interactions listed in this table d. No known comorbidity includes women who linked to hospital records and did not have comorbidity and women who did not link to a hospital record (unknown comorbidity) e. Significance of interaction terms based on likelihood ratio tests f. p = 0.9998 g. Significance of main effect based on likelihood ratio test

### All-cause mortality

All-cause survival followed similar patterns to cause-specific survival (Table 3) and mortality: higher comorbidity score was associated with higher mortality, more so for non-Indigenous than Indigenous women; excess mortality for Indigenous compared with non-Indigenous women was evident only for women without comorbidity; and mortality increased with increasing age, for women without comorbidity or score of 1, but not for women with a comorbidity score of 2+.

### Sensitivity analysis

Overall, 12.5% of women in the cohort did not link to any hospital record (Indigenous 2.0%; non-Indigenous 12.9%). Failure to link was less common for women from jurisdictions with private hospital data (5.8%) than without (25.5%), and less common with increasing socioeconomic disadvantage, more so in the jurisdictions without private hospital data (least disadvantaged areas 28%, most disadvantaged areas 6%) than in those with private hospital data (7.7%, 4.4%). Women in the jurisdictions with private hospital data had similar comorbidity as those in the jurisdictions without private hospital data (11% vs. 10%). Cause-specific mortality followed similar patterns regardless of whether women who did not link to any hospitalisation were included in the analysis (and assumed to have no comorbidity) or excluded.

### Stage at diagnosis

**Fig 2:**
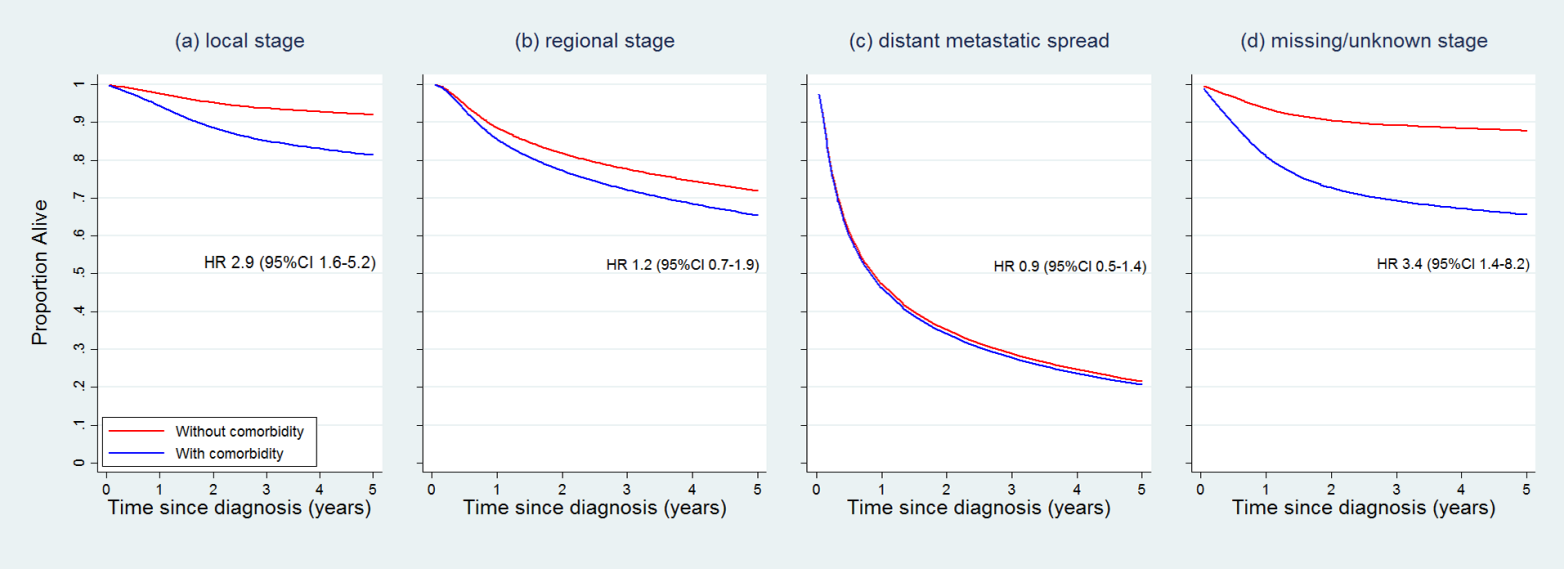
Cause-specific survival for NSW women (n = 1,069) by Charlson Comorbidity Index score, stratified by cancer stage, adjusted for age at diagnosis and Indigenous status.

Almost half of NSW women (47.0%) were diagnosed with localised cancer (16.4% had unknown or missing stage). The odds of advanced-stage diagnosis were greater for Indigenous than non-Indigenous women (OR 1.9, 95%CI 0.99–3.5) and for women with comorbidity (score 1: OR 3.0, 1.8–5.1; score 2+: OR 2.1, 1.4–3.2) than those without. In the multivariable analysis (adjusted for Indigenous status, age at diagnosis, histology type, and socioeconomic status) cause-specific mortality was higher for women with than without comorbidity (score 1: HR 1.7, 1.1–2.7; 2+: HR 1.8, 1.3–2.6), but these associations were attenuated and became not statistically significant when also adjusted for stage at diagnosis (score 1: HR 1.3, 0.8–2.0; 2+: HR 1.2, 0.9–1.8). Stratified survival curves show that comorbidity is associated with lower survival only for women with localised disease (and for women with unknown stage), but not for women with regional or distant spread (Fig 2).

## Discussion

We have shown that increased comorbidity is associated with reduced five-year cause-specific and all-cause survival for Australian women diagnosed with cervical cancer. This finding is consistent with previous findings from New Zealand,[11] a neighbouring country with similar culture, healthcare system and cervical screening program,[36, 37] but is not consistent with findings reported from other countries.[12, 13] Elsewhere and for other cancer types, it has been well established that comorbidity is associated with higher mortality from any cause, but it is less clear if comorbidity is associated with cancer-specific death.[2] One explanation for our results is that comorbidity may form a barrier to participation in cancer screening,[38] which may lead to more advanced disease at diagnosis.[39] Comorbidity may also limit treatment options and increase the risk of treatment complications.[1] We were unable to assess screening participation and treatment in this study. However, for NSW women, we were able to demonstrate that comorbidity is associated with more advanced disease at diagnosis, which is suggestive of lower screening participation by these women.

Surprisingly, the pattern of increasing mortality with increasing comorbidity was only observed for non-Indigenous women. For Indigenous women, the relationship between comorbidity and mortality was less definitive. While this is in part due to the small numbers of Indigenous women, which leads to low precision and power, the results suggest a much weaker, if any, association between comorbidity and cervical cancer mortality for Indigenous women. Adjustment for comorbidity, particularly as a summary measure rather than individual conditions, did reduce the Indigenous: non-Indigenous hazard ratio somewhat: 18% with the Charlson summary score and 9% with individual Charlson conditions. A similar pattern was observed when the Elixhauser score was used. However, these reductions were not statistically significant (as indicated by overlapping confidence intervals) and there still remained substantial excess mortality for Indigenous women compared to non-Indigenous women after adjustment.

Indigenous women with comorbidity score 2+ only had slightly higher mortality than Indigenous women without comorbidity and survival was highest for Indigenous women with a comorbidity score of 1. In Queensland, most Indigenous women do not participate in cervical screening, but those who do screen tend to do so regularly.[21] It is plausible that these are the women with mild comorbidity, who may have greater contact with the healthcare system and thus may be more likely to receive opportunistic screening or an earlier diagnosis of symptomatic disease.

Differences in treatment receipt for Indigenous and non-Indigenous women may also explain this finding. For NSW non-small cell lung cancer patients, Indigenous patients without comorbidity were half as likely to receive surgery as non-Indigenous patients, while there was no difference in surgery rates for Indigenous and non-Indigenous patients with comorbidity.[40] Although it is not clear whether these patterns are generalizable to cervical cancer treatment, the combination of lower participation in cervical screening,[21] more advanced disease at diagnosis,[22, 41, 42] and less optimal cancer treatment[23] is likely to at least partially explain the survival differential for Indigenous women compared to non-Indigenous women. That this relationship is restricted to women without comorbidity suggests factors other than those measured in this study are responsible for most of the survival differential and this warrants further investigation.

Australia has transitioned its National Cervical Screening Program from two-yearly Pap testing to the more effective Human Papillomavirus screening test with a longer screening interval of five years.[16] It is not clear what effect this change will have on screening participation. The longer interval may improve screening participation because it is less of a burden on women, or alternatively participation may fall because infrequency leads to disengagement with the program, particularly for women without comorbidity who have less reason to interact with the healthcare system. The new program will also provide an option of in-clinic self-collected screening (a vaginal swab) for hard-to-reach and under-screened women.[16] While this is a valuable option, if comorbidity prevents women from attending a medical centre in the first place, it may have little impact on screening participation and cervical cancer outcomes for these women. Understanding how comorbidity influences women’s participation in the screening program may provide valuable insight as to whether outreach services are required (e.g. self-sampling in the home setting under the supervision of a health care worker).

Geographical remoteness and socioeconomic disadvantage could impact mortality by limiting access to preventive (e.g. screening) or treatment services. In our study, living in higher socioeconomic areas was associated with higher survival, similar to that observed in previous studies,[43, 44] and Indigenous women living in areas of greater disadvantage accounted for some of the Indigenous: non-Indigenous survival differential. In contrast with previous findings,[19, 45] place of residence was not associated with excess mortality after adjustment for other factors. Survival inequalities previously reported for women in remote areas may partly reflect the higher proportion of Indigenous people and socioeconomic disadvantage in these areas.[44, 46]

### Strengths and limitations

The relationship of increasing mortality with increasing comorbidity is clear for non-Indigenous women. However, for Indigenous women the small numbers in some groups, especially the group with comorbidity score of 1, meant the confidence intervals were wide and findings imprecise. Our findings do not provide strong evidence that there is no association between comorbidity and excess mortality for Indigenous women, but our findings do indicate that if there is such an association for Indigenous women, it is considerably smaller than for non-Indigenous women. The larger numbers of women with no comorbidity provide strength to our finding of an important Indigenous: non-Indigenous survival differential among women without comorbidity. This finding suggests that factors other than comorbidity are responsible for the disparity in cervical cancer survival between Indigenous and other Australian women. This study examined the relationship between individual comorbid conditions and cause-specific survival for the whole cohort of women, but the small number of Indigenous women meant this could not be explored separately for them. For the same reason, the experience of Torres Strait Islander women could not be investigated separately from that of Aboriginal women.

A major limitation of this study was the lack of information on women’s cervical screening, cancer treatment, and stage of cancer at diagnosis. Cancer stage is a powerful predictor of survival and thus its absence from cancer registries limits evaluation of cancer programs, the effective allocation of resources, and the ability to accurately compare cancer outcomes across population groups.[47] In Australia, stage at diagnosis is only routinely collected by one registry, however a previous validation study found that the routine collection of staging information by cancer registries would be feasible for most cancer types;[47] we urge them to do so.

This study relies on: (1) the ability of the comorbidity tool to capture all important comorbidity for women with cervical cancer; (2) the accuracy and completeness of hospitals’ coded diagnosis data; and (3) accurate record linkage. We utilised two measures of comorbidity, and while the Elixhauser (which includes a greater number of conditions) identified more comorbidity, the patterns of association between comorbidity and mortality were similar for the two measures. Neither the Charlson nor the Elixhauser indices were developed or have been validated for Indigenous people with cancer, despite their common use for this population group in Australia. Given the different characteristics of Indigenous women compared to non-Indigenous women (e.g. more likely to be diagnosed at a younger age and with advanced-stage disease) it is possible that neither of these indices capture the most important comorbidities for this population. Development and use of an Indigenous-specific comorbidity index may find an association between comorbidity and excess mortality that was not apparent in this study. We limited our definition of comorbidity to conditions recorded in inpatient records for the two years prior to cervical cancer diagnosis. This means that included conditions may have been diagnosed at any time in the past. While a longer lookback period may ascertain more comorbid conditions, previous studies suggest a longer lookback is unlikely to alter the relationship between comorbidity and mortality.[11, 48, 49]

It is likely that we did not identify all comorbid conditions, particularly conditions that are managed in primary care and do not warrant hospitalisation. A Canadian study demonstrated that more comorbidity was identified for hypertensive patients using hospital and health insurance data than hospital data alone (even with a longer lookback period).[50] However, while multiple data sources were found to produce a comorbidity measure that better predicted one-year mortality, the relationship between comorbidity and mortality was similar regardless of the data sources used.

Women were more likely to link to a hospital record in the jurisdictions that provided both public and private hospital data. Even when assuming those who did not link had no comorbidity, the proportion of women with comorbidity was similar in jurisdictions with and without private hospital data. Other reasons for non-linkage may include a failure of the linkage process through false negative matches, non-linkage of women who were hospitalised interstate, or true non-matches for women who had not been hospitalised. It is unknown to what degree and in which direction the lack of linkage may have biased the association between comorbidity and survival, although the sensitivity analysis suggests it had little impact.

Our comparison of cause-specific and relative survival for Indigenous and non-Indigenous women (not stratified by comorbidity level) found that the cause-specific method may underestimate five-year survival by up to 2% in comparison to relative survival. Previous research has shown that use of cause-specific and relative survival produced similar estimates for Indigenous and non-Indigenous Australians with cancer.[20] The accurate attribution of cause of death may be more difficult for cancer patients who also have other chronic conditions, including other cancers, but we have no evidence as to the direction or magnitude of this potential bias. We did not conduct a competing risks analysis for this cohort, which provides a measure of cancer mortality, taking non-cancer deaths (competing risks) into account. There is evidence that relative survival, cause-specific survival, and competing risks analysis similarly highlight the excess cancer mortality burden faced by Indigenous cancer patients compared with non-Indigenous patients.[51]

### Conclusion

Comorbidity is an important prognostic factor for Australian women diagnosed with cervical cancer, and further research (which preferably utilises multiple public health datasets to measure comorbidity) should investigate the underlying mechanisms of this relationship. Comorbidity contributed little, if any, to the lower survival for Indigenous women. There was a large survival disparity observed among those without comorbidity. Plausibly, these women have less interaction with the healthcare system and are thus less engaged with cancer screening programs and/or treatment services, and there is a role here for primary care providers to devise ways in which Indigenous women who are relatively healthy are engaged with these services and programs.

## Supporting information

S1 Table Prevalence of Elixhauser comorbidity (%) for Australian Indigenous and non-Indigenous women aged 22–89 years at diagnosis of cervical cancer, 2003-2012^a^. (DOCX)

S2 Table Crude 5-year Kaplan-Meier survival estimates for 4,467 Australian women (22–89 years)^a^ diagnosed with cervical cancer, 2003–2012, by Elixhauser comorbidity score (0,1,2+). (DOCX)

S3 Table Excess cause-specific mortality for Indigenous women compared to non-Indigenous women with cervical cancer, 2003–2012. (DOCX)

S4 Table Hazard ratios for five-year cause-specific mortality for Australian women, 22–89 years, diagnosed with cervical cancer, 2003–2012 ^a, b^. (DOCX)

S5 Table Five-year cause-specific survival estimates and hazard ratios^a^ for individual Elixhauser conditions for Australian women (n = 4,467) diagnosed with cervical cancer, 2003–2012. (DOCX)

S6 Table Hazard ratios for five-year cause-specific mortality for Australian women, 22–89 years, diagnosed with cervical cancer, 2003-2012^a^. (DOCX)

S7 Table Five-year cause-specific survival estimates and hazard ratios^a^ for individual Charlson conditions for Australian women (n = 4,467) diagnosed with cervical cancer, 2003–2012. (DOCX)
