# Supplementary material for: Comorbidity and cervical cancer survival of Indigenous and non-Indigenous Australian women: A semi-national registry-based cohort study (2003-2012)
Source: PLoS One. 2018 May 8;13(5):e0196764. doi: 10.1371/journal.pone.0196764 (PMC5940188; doi:10.1371/journal.pone.0196764)
Supplement: S1 Table — (DOCX) [file pone.0196764.s001.docx]

**Table S1: Prevalence of Elixhauser comorbidity (%) for Australian Indigenous and non-**

|  | | | | | **Indigenous**  **(n=198)** | **Non-Indigenous**  **(n=4,269)** | ***P*** |
| --- | --- | --- | --- | --- | --- | --- | --- |
| Elixhauser condition ^a^ | | | | | **%** | **%** |  |
|  | Congestive heart failure | | | | 4.0 | 0.9 | <0.001 |
|  | Cardiac arrhythmias | | | | 5.1 | 2.0 | 0.004 |
|  | Valvular disease | | | | 1.5 | 0.5 | 0.065 |
|  | Pulmonary circulation disorders | | | | 1.0 | 0.7 | 0.616 |
|  | Peripheral vascular disorders | | | | 0.5 | 0.5 | 0.941 |
|  | Hypertension, uncomplicated | | | | 11.1 | 5.1 | <0.001 |
|  | Hypertension, complicated | | | | 0.0 | 0.1 | 0.667 |
|  | Paralysis | | | | 1.5 | 0.4 | 0.028 |
|  | Other neurological disorders | | | | 2.0 | 0.5 | 0.005 |
|  | Chronic pulmonary disease | | | | 5.6 | 1.8 | <0.001 |
|  | Diabetes, uncomplicated | | | | 4.0 | 1.2 | 0.001 |
|  | Diabetes, complicated | | | | 7.6 | 2.3 | <0.001 |
|  | Hypothyroidism | | | | 2.0 | 0.2 | <0.001 |
|  | Renal failure | | | | 5.6 | 1.7 | <0.001 |
|  | Liver disease | | | | 2.5 | 0.7 | 0.006 |
|  | Peptic ulcer disease, excl. bleeding | | | | 0.0 | 0.1 | 0.709 |
|  | AIDS/HIV | | | | 0.0 | 0.0 | - |
|  | Lymphoma | | | | 0.0 | 0.1 | 0.667 |
|  | Metastatic cancer, excluding gynaecological | | | | 3.0 | 1.5 | 0.097 |
|  | Solid tumour without metastasis, excluding gynaecological | | | | 3.5 | 1.6 | 0.038 |
|  | Rheumatoid arthritis/collagen vascular diseases | | | | 0.5 | 0.4 | 0.771 |
|  | Coagulopathy | | | | 1.0 | 0.6 | 0.451 |
|  | Obesity | | | | 3.5 | 1.2 | 0.004 |
|  | Weight loss | | | | 2.0 | 0.6 | 0.021 |
|  | Fluid and electrolyte disorders | | | | 9.1 | 3.7 | <0.001 |
|  | Blood loss anaemia | | | | 3.5 | 1.3 | 0.008 |
|  | Deficiency anaemia | | | | 6.1 | 1.2 | <0.001 |
|  | Alcohol abuse | | | | 12.6 | 0.9 | <0.001 |
|  | Drug abuse | | | | 3.5 | 0.6 | <0.001 |
|  | Psychoses | | | | 3.0 | 0.4 | <0.001 |
|  | Depression | | | | 5.1 | 1.2 | <0.001 |
|  |  |  |  |  |  |  |  |
| Elixhauser score^a^ | | | | |  |  |  |
|  | 0 (No known comorbidity) ^c^ | | | | 54.6 | 83.4 | <0.001 |
|  | 1 | | | | 20.2 | 8.6 |  |
|  | 2 | | | | 8.6 | 3.7 |  |
|  | 3 | | | | 7.6 | 1.9 |  |
|  | 4+ | | | | 9.1 | 2.4 |  |

**Indigenous women aged 22-89 years at diagnosis of cervical cancer, 2003-2012^a^**

NOTES:

1. Comorbidity was measured using diagnoses codes contained in hospital admissions data for the two years prior and including the woman’s cervical cancer diagnosis date.
2. These categories excluded gynaecological cancers.
3. No known comorbidity includes women who linked to hospital records and did not have comorbidity and women who did not link to a hospital record and have unknown comorbidity.
