# Supplementary material for: Comorbidity and cervical cancer survival of Indigenous and non-Indigenous Australian women: A semi-national registry-based cohort study (2003-2012)
Source: PLoS One. 2018 May 8;13(5):e0196764. doi: 10.1371/journal.pone.0196764 (PMC5940188; doi:10.1371/journal.pone.0196764)
Supplement: S2 Table — (DOCX) [file pone.0196764.s002.docx]

**Table S2: Crude 5-year Kaplan-Meier survival estimates for 4,467 Australian women (22-89 years)^a^ diagnosed with cervical cancer,2003-2012, by Elixhauser comorbidity score (0,1,2+)**

|  | | **Five-year *cause-specific* survival (%, 95%CI)** | | | |
| --- | --- | --- | --- | --- | --- |
|  | | **All women** | **Elixhauser comorbidity score ^b^** | | |
|  | |  | **0** | **1** | **2+** |
| All women | | 75.8 (74.4-77.2) | 80.8 (79.4-82.2) | 59.9 (45.6-64.8) | 43.2 (37.4-48.8) |
| Indigenous status | |  |  |  |  |
|  | non-Indigenous | 76.6 (75.1-77.9) | 81.3 (79.9-82.7) | 58.7 (53.1-64.0) | 43.8 (37.5-49.8) |
|  | Indigenous | 60.2 (52.4-67.0) | 65.4 (55.0-73.9) | 70.3 (52.5-82.5) | 40.3 (25.1-55.0) |
| Age group ^a^ | |  |  |  |  |
|  | youngest-39 years | 88.9 (87.0-90.5) | 90.4 (88.6-92.0) | 78.7 (68.0-86.2) | 53.7 (35.5-68.8) |
|  | 40-49 years | 81.3 (78.5-83.7) | 84.7 (81.9-87.1) | 63.6 (52.4-72.8) | 54.4 (39.1-67.5) |
|  | 50-59 years | 70.2 (66.5-73.6) | 75.2 (71.2-78.7) | 51.8 (39.3-62.9) | 49.6 (35.7-62.0) |
|  | 60-69 years | 63.4 (58.8-67.6) | 68.8 (63.7-73.3) | 64.0 (49.4-75.4) | 25.5 (14.5-38.0) |
|  | 70-79 years | 57.9 (51.3-64.0) | 64.2 (55.8-71.5) | 42.2 (25.2-58.2) | 51.5 (38.0-63.4) |
|  | 80-89 years | 38.2 (31.1-45.3) | 44.3 (34.8-53.3) | 38.8 (21.1-56.2) | 23.8 (12.4-37.2) |
| Diagnostic period | |  |  |  |  |
|  | 2003-2007 | 75.6 (73.8-77.3) | 81.1 (79.3-82.8) | 58.1 (51.5-64.2) | 44.2 (37.0-51.1) |
|  | 2008-2012 | 76.3 (73.8-78.6) | 80.6 (78.0-82.9) | 62.6 (53.1-70.8) | 42.0 (32.3-51.4) |
|  |  |  |  |  |  |
|  |  | **Five-year *all-cause* survival (%, 95%CI)** | | | |
|  | | **All women** | **Elixhauser comorbidity score ^b^** | | |
|  | |  | **0** | **1** | **2+** |
| All women | | 70.8 (69.4-72.3) | 77.5 (75.9-78.9) | 52.1 (46.8-57.1) | 29.7 (24.9-34.6) |
| Indigenous status | |  |  |  |  |
|  | non-Indigenous | 71.7 (70.2-73.1) | 77.9 (76.3-79.4) | 50.8 (45.2-56.1) | 30.4 (25.2-35.8) |
|  | Indigenous | 53.4 (45.8-60.4) | 63.7 (53.5-72.3) | 63.6 (46.2-76.7) | 26.1 (14.6-39.2) |
| Age group ^a^ | |  |  |  |  |
|  | youngest-39 years | 87.6 (85.7-89.3) | 89.4 (87.5-91.1) | 77.6 (66.8-85.3) | 46.1 (28.9-61.7) |
|  | 40-49 years | 78.3 (75.4-80.9) | 81.9 (78.9-84.5) | 60.6 (49.2-70.3) | 50.0 (35.6-62.9) |
|  | 50-59 years | 65.7 (61.9-69.2) | 72.7 (68.6-76.3) | 46.3 (34.1-57.5) | 34.9 (23.8-46.2) |
|  | 60-69 years | 57.6 (53.1-61.9) | 64.3 (59.1-69.0) | 56.5 (42.3-68.5) | 16.7 (8.6-27.0) |
|  | 70-79 years | 44.9 (38.7-50.9) | 54.3 (45.9-62.0) | 27.8 (15.0-42.2) | 32.9 (22.3-44.0) |
|  | 80-89 years | 21.3 (16.1-27.0) | 29.5 (21.7-37.7) | 18.5 (8.3-31.9) | 6.2 (1.6-15.4) |
| Diagnostic period | |  |  |  |  |
|  | 2003-2007 | 70.3 (68.4-72.1) | 77.6 (75.7-79.4) | 50.5 (44.0-56.6) | 29.5 (23.7-35.5) |
|  | 2008-2012 | 72.1 (69.5-74.5) | 77.7 (74.9-80.2) | 54.9 (45.3-63.5) | 28.6 (23.7-40.3) |
|  |  |  |  |  |  |

*Abbreviations: CI: Confidence Intervals*

NOTES:

1. All jurisdictions provided data for women aged 22-89 years at diagnosis of cervical cancer, with the exception of Queensland, which only provided data on women aged 22-69 years at diagnosis.
2. No known comorbidity includes women who linked to hospital records and did not have comorbidity and women who did not link to a hospital record and have unknown comorbidity.
