# Supplementary material for: Comorbidity and cervical cancer survival of Indigenous and non-Indigenous Australian women: A semi-national registry-based cohort study (2003-2012)
Source: PLoS One. 2018 May 8;13(5):e0196764. doi: 10.1371/journal.pone.0196764 (PMC5940188; doi:10.1371/journal.pone.0196764)
Supplement: S3 Table — (DOCX) [file pone.0196764.s003.docx]

**Table S3: Excess cause-specific mortality for Indigenous women compared to non-Indigenous women with cervical cancer, 2003-2012**

| **Adjusted for:** | | | **HR (95%CI)^a^** |
| --- | --- | --- | --- |
|  | Age at diagnosis | | 2.59 (2.03-3.31) |
|  | + histological type, &SES | | 2.20 (1.71-2.81) |
|  |  | (a) +Elixhauser score | 1.56 (1.21-2.02) |
|  |  | (b) + individual conditions^b^ | 1.77 (1.35-2.31) |
| **Stratified by Elixhauser score:**^c^ | | |  |
|  | Score 0 ^d^ | | 2.84 (2.00-4.03) |
|  | Score 1 | | 0.74 (0.40-1.40) |
|  | Score ≥2 | | 1.00 (0.69-4.54) |

*Abbreviations: HR: Hazard Ratio; SES: socioeconomic status (quintile 1-5)*

NOTES:

1. Hazard ratio for Indigenous women compared to non-Indigenous women (reference group)
2. 20 conditions included in the Elixhauser score that were associated with mortality in a multivariable model adjusted for age at diagnosis, Indigenous status, histology type and socioeconomic status (HR >1.5).
3. Adjusted for age at diagnosis, histology type and socioeconomic status.
4. No known comorbidity includes women who linked to hospital records and did not have comorbidity and women who did not link to a hospital record and have unknown comorbidity.
