# Supplementary material for: Comorbidity and cervical cancer survival of Indigenous and non-Indigenous Australian women: A semi-national registry-based cohort study (2003-2012)
Source: PLoS One. 2018 May 8;13(5):e0196764. doi: 10.1371/journal.pone.0196764 (PMC5940188; doi:10.1371/journal.pone.0196764)
Supplement: S4 Table — (DOCX) [file pone.0196764.s004.docx]

**Table S4: Hazard ratios for five-year cause-specific mortality for Australian women, 22-89 years, diagnosed with cervical cancer, 2003-2012 ^a, b^**

|  |  | Adjusted HR ^c^ | 95%CI |
| --- | --- | --- | --- |
| **Elixhauser comorbidity score** | |  |  |
| *Indigenous women* | |  |  |
|  | 0 (No known comorbidity)^d^ | 1.00 |  |
|  | 1 | 0.74 | (0.37-1.45) |
|  | 2+ | 1.47 | (0.88-2.45) |
| *Non-Indigenous women* | |  |  |
|  | 0 (No known comorbidity)^d^ | 1.00 |  |
|  | 1 | 2.81 | (2.24-3.53) |
|  | 2+ | 4.16 | (3.25-5.33) |
| **Age at diagnosis, per year increase** | |  |  |
|  | Elixhauser score 0 ^d^ | 1.04 | (1.04-1.05) |
|  | Elixhauser score 1 | 1.02 | (1.01-1.03) |
|  | Elixhauser score ≥2 | 1.02 | (1.00-1.03) |
| **Area level socioeconomic disadvantage** | |  |  |
|  | Most advantaged (Q5) | 1.00 |  |
|  | Q4 | 0.98 | (0.80-1.21) |
|  | Q3 | 1.14 | (0.94-1.39) |
|  | Q2 | 1.31 | (1.05-1.63) |
|  | Most disadvantaged (Q1) | 1.50 | (1.19-1.88) |
|  | Missing | 0.83 | (0.42-1.64) |
| **Histology type** | |  |  |
|  | Squamous cell carcinoma | 1.00 |  |
|  | Adenocarcinoma | 0.79 | (0.66-0.94) |
|  | Adeno-squamous carcinoma | 1.27 | (0.90-1.81) |
|  | Other carcinoma or sarcoma | 2.24 | (1.86-2.71) |

*Abbreviations: HR: hazard ratio; CI: confidence interval; Q: quintile.*

Notes:

1. All states/territories entered the study between January and July 2003, except for July which entered the study in January 2007. States/territories exited the study in December 2007 (NSW), December 2009 (QLD), December 2010 (NT), December 2011 (WA), and December 2012 (VIC and SA).
2. All states/territories contributed women aged 22-89 years at diagnosis, except for Queensland which contributed women aged 22-69 years only.
3. The hazard ratios were mutually adjusted for all variables listed in this table and the interactions between comorbidity and survival time and comorbidity and age at diagnosis.
4. No known comorbidity includes women who linked to hospital records and did not have comorbidity and women who did not link to a hospital record and have unknown comorbidity.
