# Supplementary material for: Comorbidity and cervical cancer survival of Indigenous and non-Indigenous Australian women: A semi-national registry-based cohort study (2003-2012)
Source: PLoS One. 2018 May 8;13(5):e0196764. doi: 10.1371/journal.pone.0196764 (PMC5940188; doi:10.1371/journal.pone.0196764)
Supplement: S5 Table — (DOCX) [file pone.0196764.s005.docx]

**Table S5: Five-year cause-specific survival estimates and hazard ratios^a^ for individual Elixhauser conditions for Australian women (n=4,467) diagnosed with cervical cancer, 2003-2012**

| **Elixhauser comorbidity^b^** | **5-year KM**  **survival probability** | **Separate Models^c^** | **Combined Model^d^** |
| --- | --- | --- | --- |
|  |  | **HR (95%CI)** | **HR (95%CI)** |
| No known comorbidity^e^ | 80.8 (79.4-82.2) |  |  |
| Congestive heart failure | 41.7 (24.9-57.7) | 1.44 (0.94-2.21) |  |
| Cardiac arrhythmias | 48.2 (36.9-58.6) | 1.29 (0.94-1.77) |  |
| Valvular disease | 56.8 (32.5-75.2) | 0.77 (0.39-1.51) |  |
| Pulmonary circulation disorders | 34.6 (14.9-55.2) | 2.00 (1.22-3.29) | 1.39 (0.81-2.41) |
| Peripheral vascular disorders | 64.2 (36.1-82.5) | 0.64 (0.29-1.45) |  |
| Hypertension, uncomplicated | 46.4 (38.7-53.8) | 1.40 (1.13-1.73) | 0.78 (0.59-1.04) |
| Paralysis | 22.9 (1.8-58.3) | 1.73 (0.95-3.15) | 1.22 (0.63-2.37) |
| Other neurological disorders | 41.2 (18.0-63.2) | 1.88 (1.06-3.35) | 1.09 (0.58-2.04) |
| Chronic pulmonary disease | 64.3 (51.1-74.8) | 0.91 (0.61-1.35) |  |
| Diabetes, uncomplicated | 48.2 (34.3-60.8) | 1.95 (1.33-2.84) | 1.38 (0.90-2.10) |
| Diabetes, complicated | 42.6 (31.5-53.2) | 1.63 (1.22-2.16) | 1.07 (0.73-1.56) |
| Hypothyroidism | 52.9 (22.6-76.1) | 1.34 (0.59-3.03) |  |
| Renal failure | 28.8 (18.1-40.5) | 3.05 (2.29-4.07) | 1.88 (1.32-2.68) |
| Liver disease | 61.9 (43.3-76.0) | 1.73 (0.99-3.00) | 0.82 (0.46-1.49) |
| Metastatic cancer, excl. gynaecological | 16.1 (3.1-30.3) | 5.61 (4.17-7.55) | 5.05 (3.61-7.04) |
| Solid tumour without metastasis, excl. gynaecological | 41.9 (26.7-56.5) | 1.55 (1.09-2.22) | 0.92 (0.62-1.37) |
| Rheumatoid arthritis/collagen vascular diseases | 48.3 (22.1-70.4) | 1.69 (0.84-3.40) | 1.24 (0.60-2.56) |
| Coagulopathy | 37.2 (16.7-57.9) | 1.72 (1.00-2.97) | 1.24 (0.67-2.30) |
| Obesity | 70.4 (55.3-81.3) | 1.05 (0.63-1.76) |  |
| Weight loss | 22.1 (7.5-41.5) | 4.87 (3.14-7.56) | 3.54 (2.22-5.65) |
| Fluid and electrolyte disorders | 38.7 (30.5-46.8) | 2.82 (2.27-3.51) | 1.94 (1.49-2.52) |
| Blood loss anaemia | 44.2 (30.8-56.7) | 3.14 (2.20-4.48) | 2.16 (1.48-3.14) |
| Deficiency anaemia | 46.7 (32.9-59.3) | 2.39 (1.66-3.42) | 1.64 (1.11-2.41) |
| Alcohol abuse | 54.5 (40.1-66.8) | 2.22 (1.46-3.38) | 1.27 (0.79-2.06) |
| Drug abuse | 71.7 (52.3-84.2) | 1.50 (0.77-2.92) | 0.98 (0.48-2.00) |
| Psychoses | 51.3 (29.0-69.8) | 2.21 (1.21-4.04) | 1.95 (1.03-3.68) |
| Depression | 54.0 (40.1-66.1) | 2.12 (1.43-3.16) | 1.58 (1.03-2.18) |

*Abbreviations: KM: Kaplan Meier; HR: Hazard Ratio; CI: Confidence Interval;*

NOTES:

1. Reference group is those without the individual comorbidity.
2. None of the women in this cohort had a co-diagnosis of AIDS/HIV, and fewer than five women had a co-diagnosis of hypertension, with complications; peptic ulcer, excluding bleeding; and lymphoma. Therefore each of these conditions were excluded from this table.
3. A separate model was fitted for each individual condition, adjusted for age at diagnosis, Indigenous status, histology type and socioeconomic status.
4. A single model was fitted , adjusted for age at diagnosis, Indigenous status, histology type, socioeconomic status, and each Charlson condition that had an HR >1.5 or were statistically significant in the corresponding separate model (note c)
5. No known comorbidity includes women who linked to hospital records and did not have comorbidity and women who did not link to a hospital record and have unknown comorbidity.
