# Supplementary material for: Comorbidity and cervical cancer survival of Indigenous and non-Indigenous Australian women: A semi-national registry-based cohort study (2003-2012)
Source: PLoS One. 2018 May 8;13(5):e0196764. doi: 10.1371/journal.pone.0196764 (PMC5940188; doi:10.1371/journal.pone.0196764)
Supplement: S6 Table — (DOCX) [file pone.0196764.s006.docx]

**Table S6: Hazard ratios for five-year cause-specific mortality for Australian women, 22-89 years, diagnosed with cervical cancer, 2003-2012^a^**

|  | | **Model 1** | **Model 2** | **Model 3** | **Model 4** |
| --- | --- | --- | --- | --- | --- |
|  | | Including terms for Indigenous status and age | Model 1 plus SES^b^, histology type | Model 2 plus comorbidity score | Model 2 plus comorbid conditions |
| Indigenous status | |  |  |  |  |
|  | Non-Indigenous | 1.0 | 1.0 | 1.0 | 1.0 |
|  | Indigenous | 2.6 (2.0-3.3) | 2.2 (1.7-2.8) | 1.8 (1.4-2.3) | 2.0 (1.5-2.6) |
| Per year increase in  age at diagnosis | | 1.04 (1.04-1.05) | 1.04 (1.04-1.01) | 1.04 (1.03-1.04) | 1.04 (1.03-1.04) |
| Socioeconomic disadvantage^b^ | |  |  |  |  |
|  | Most advantaged (Q5) | NA | 1.0 | 1.0 | 1.0 |
|  | Q4 | NA | 1.0 (0.8-1.2) | 1.0 (0.8-1.2) | 0.9 (0.8-1.2) |
|  | Q3 | NA | 1.2 (1.0-1.4) | 1.2 (1.0-1.4) | 1.1 (0.9-1.4) |
|  | Q2 | NA | 1.4 (1.1-1.7) | 1.4 (1.1-1.7) | 1.3 (1.1-1.6) |
|  | Most disadvantaged (Q1) | NA | 1.6 (1.3-2.0) | 1.6 (1.2-2.0) | 1.5 (1.2-1.9) |
|  | Missing | NA | 0.8 (0.4-1.5) | 0.9 (0.4-1.7) | 0.8 (0.4-1.5) |
| Histology type | |  |  |  |  |
|  | Squamous cell carcinoma | NA | 1.0 | 1.0 | 1.0 |
|  | Adenocarcinoma | NA | 0.8 (0.6-0.9) | 0.8 (0.6-0.9) | 0.8 (0.6-0.9) |
|  | Adeno-squamous carcinoma | NA | 1.2 (0.8-1.7) | 1.2 (0.9-1.8) | 1.3 (0.9-1.8) |
|  | Other carcinoma or sarcoma | NA | 2.2 (1.8-2.6) | 2.1 (1.7-2.5) | 2.2 (1.8-2.7) |
| Charlson comorbidity score | |  |  |  |  |
|  | Score 0 | NA | NA | 1.0 | NA |
|  | Score 1 | NA | NA | 1.3 (0.9-1.7) | NA |
|  | Score 2+ | NA | NA | 2.5 (2.1-3.0) | NA |
| Charlson condition^c,d^ | |  |  |  |  |
|  | Cerebrovascular disease | NA | NA | NA | 1.2 (0.6-2.4) |
|  | Dementia | NA | NA | NA | 2.1 (1.2-3.5) |
|  | Connective tissue disease | NA | NA | NA | 1.2 (0.6-2.4) |
|  | Mild liver disease | NA | NA | NA | 1.3 (0.6-2.6) |
|  | Diabetes w/o complications | NA | NA | NA | 1.3 (0.9-2.0) |
|  | Diabetes with complication | NA | NA | NA | 1.0 (0.7-1.4) |
|  | Paraplegia/hemiplegia | NA | NA | NA | 1.4 (0.5-3.4) |
|  | Mod-severe renal disease | NA | NA | NA | 2.5 (1.8-3.4) |
|  | Mod-severe liver disease | NA | NA | NA | 2.2 (0.7-7.3) |
|  | Metastatic cancer | NA | NA | NA | 5.3 (3.9-7.2) |

*NOTES:*

1. Three separate flexible parametric regression models, containing terms for variables listed. NA, not applicable.
2. Area-level socioeconomic disadvantage
3. 10 conditions in the Charlson comorbidity index that were found to be associated with cause-specific mortality (HR>1.5) when entered into the model one at a time, adjusted for age at diagnosis, Indigenous status, area-level socioeconomic status, and histology type.
4. Reference group for each condition is those without that condition
