# Supplementary material for: Comorbidity and cervical cancer survival of Indigenous and non-Indigenous Australian women: A semi-national registry-based cohort study (2003-2012)
Source: PLoS One. 2018 May 8;13(5):e0196764. doi: 10.1371/journal.pone.0196764 (PMC5940188; doi:10.1371/journal.pone.0196764)
Supplement: S7 Table — (DOCX) [file pone.0196764.s007.docx]

| **Charlson comorbidity ^b^** | **5-year KM**  **survival probability** | **Separate Models^c^** | **Combined Model^d^** |
| --- | --- | --- | --- |
|  |  | **HR (95%CI)** | **HR (95%CI)** |
| No known comorbidity^e^ | 79.0 (77.6-80.3) |  |  |
| Acute myocardial infarction | 72.0 (49.6-85.7) | 0.77 (0.36-1.62) |  |
| Congestive heart failure | 41.7 (24.9-57.7 | 1.44 (0.94-2.21) |  |
| Peripheral vascular disease | 64.2 (36.1-82.5) | 0.64 (0.29-1.45) |  |
| Cerebrovascular disease^f^ | - | 1.73 (1.05-2.87) | 1.16 (0.57-2.40) |
| Dementia | 13.1 (1.1-39.9) | 2.91 (1.73-4.91) | 2.06 (1.21-3.53) |
| Chronic pulmonary disease | 64.3 (51.1-74.8) | 0.91 (0.61-1.35) |  |
| Connective tissue disease | 48.3 (22.1-70.4) | 1.69 (0.84-3.40) | 1.18 (0.58-2.40) |
| Peptic ulcer disease | 66.7 (19.5-90.4) | 0.72 (0.18-2.89) |  |
| Mild liver disease | 64.7 (45.1-78.8) | 1.59 (0.87-2.90) | 1.25 (0.61-2.57) |
| Diabetes w/o complications | 49.5 (34.5-60.4) | 1.73 (1.25-2.41) | 1.34 (0.92-1.99) |
| Diabetes with complication | 42.1 (31.0-52.7) | 1.66 (1.25-2.21) | 1.03 (0.73-1.44) |
| Paraplegia/hemiplegia | 22.6 (1.7-58.2) | 1.55 (0.80-3.01) | 1.35 (0.53-3.43) |
| Moderate-severe renal disease | 28.8 (18.1-40.5) | 3.05 (2.89-4.07) | 2.50 (1.81-3.44) |
| Other cancers^g^ | 46.1 (30.8-60.0) | 1.30 (0.90-1.88) |  |
| Moderate-severe liver disease | 50.0 (15.2-77.5) | 2.85 (1.06-7.62) | 2.24 (0.68-7.30) |
| Metastatic cancer^g^ | 16.1 (6.1-30.3) | 5.61 (4.17-7.55) | 5.31 (3.93-7.16) |

**Table S7: Five-year cause-specific survival estimates and hazard ratios^a^ for individual Charlson conditions for Australian women(n=4,467) diagnosed with cervical cancer, 2003-2012**

*Abbreviations: KM: Kaplan-Meier; HR: Hazard Ratio; CI: Confidence Interval;*

NOTES:

1. Reference group is those without the individual comorbidity.
2. None of the women in this cohort had a co-diagnosis of AIDS/HIV and thus this condition is not included in the table.
3. A separate model was run for each individual condition, adjusted for age at diagnosis, Indigenous status, histology type and socioeconomic status.
4. A single model was run, adjusted for age at diagnosis, Indigenous status, histology type, socioeconomic status, and each Charlson condition that had an HR >1.5in the corresponding separate model (note c)
5. No known comorbidity includes women who linked to hospital records and did not have comorbidity and women who did not link to a hospital record and have unknown comorbidity.
6. The Kaplan-Meier survival probability could not be calculated for cerebrovascular disease as none of the women with this condition survived five years past their diagnosis of cervical cancer.
7. Gynaecological cancers were excluded from the ‘other cancers’ and ‘metastatic cancer” categories.
